# A Case Report of Malakoplakia of the Gallbladder: A Diagnostic Challenge and Pathological Surprise

**DOI:** 10.7759/cureus.69257

**Published:** 2024-09-12

**Authors:** Aayush Lakkanna, Lileswar Kaman, Niladri Mohan Raypattanaik, Naveen Pentakota, Aravind Sekar

**Affiliations:** 1 General Surgery, Postgraduate Institute of Medical Education and Research, Chandigarh, IND; 2 Histopathology, Postgraduate Institute of Medical Education and Research, Chandigarh, IND

**Keywords:** adenocarcinoma of gall bladder, elevated ca 19-9, gallbladder removal, malakoplakia, surgical case report

## Abstract

Malakoplakia, a rare granulomatous inflammatory condition, typically manifests in the genitourinary system. Its occurrence in the gallbladder is exceptionally uncommon, posing significant diagnostic challenges. A 68-year-old male presented with recurrent epigastric pain, and a suspicious gallbladder mass was found on imaging. He then underwent surgery due to concerns for malignancy. Histopathology of the surgical specimen revealed malakoplakia, highlighting the diagnostic dilemma encountered in such instances.

## Introduction

Malakoplakia is a chronic granulomatous inflammatory disorder that affects many organs, like the urinary bladder (most common), kidney, stomach, gallbladder, pancreas, and lymph nodes. It is characterized by histiocytic infiltrates with eosinophilic cytoplasm and basophilic intracytoplasmic inclusions containing iron and calcium known as Michaelis-Gutmann bodies, which are pathognomonic but not essential to make the diagnosis [1]. The involvement of the gallbladder is exceptionally rare [1-3]. An immunocompetent patient presented with symptoms related to chronic cholecystitis, and an extended cholecystectomy was performed due to a suspicion of malignancy. The histopathological examination of the gallbladder revealed malakoplakia-like changes, highlighting a rare, often misdiagnosed condition affecting even immunocompetent individuals, with the unusual involvement of the gallbladder.

## Case presentation

Our case 

A 68-year-old male with a past medical history of diabetes mellitus, controlled with oral hypoglycemic agents, presented with a 9-month history of recurrent epigastric pain. The pain was colicky in nature, occurring from once every month to once every 5-10 days, with moderate to severe intensity. It did not radiate to the back and was relieved by intravenous analgesics. He also reported associated dyspepsia but no vomiting, fever, jaundice, or change in bowel habits.

Investigations

**Figure 1:**
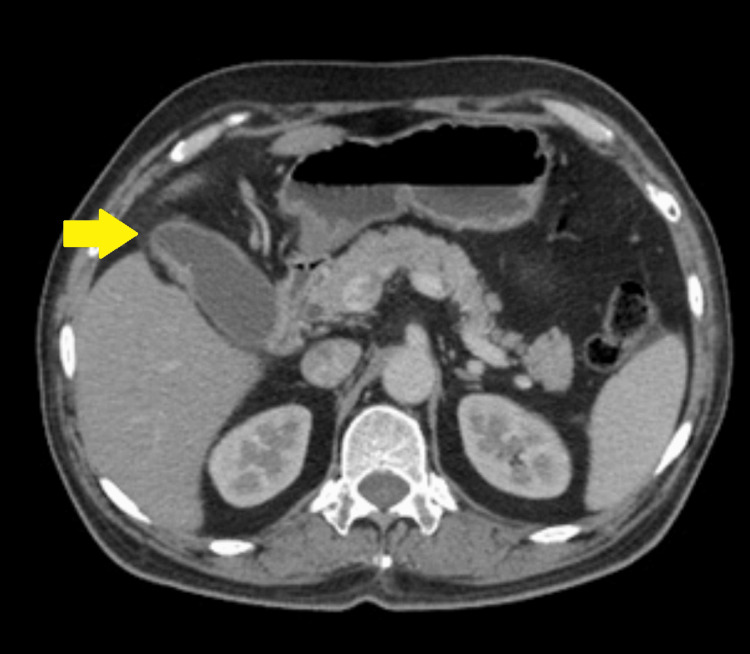
Preoperative contrast-enhanced CT of the abdomen (axial section) showing mild asymmetric mural thickening involving the gallbladder (yellow arrow). Fat planes with the liver are well maintained. Mild pericholecystic fat stranding is present. No enlarged lymph nodes are found.

**Figure 2:**
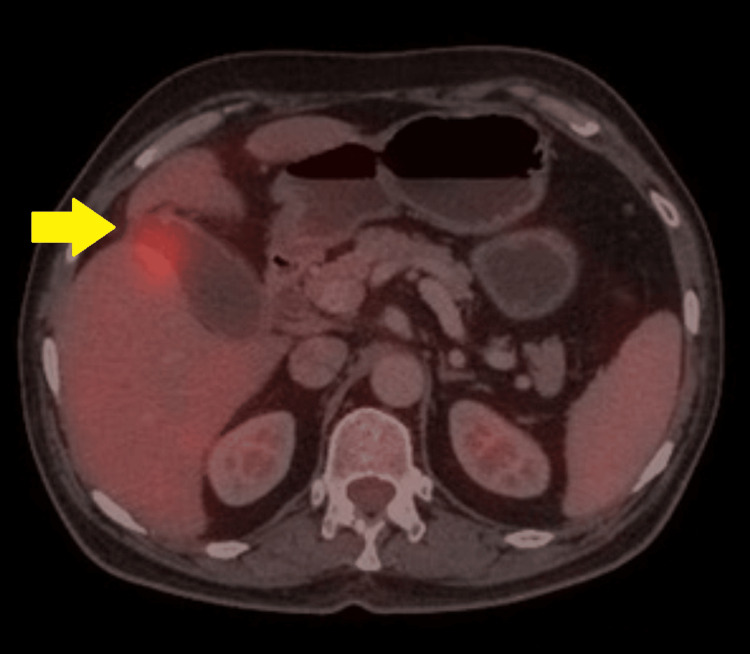
Preoperative FDG-PET CT (axial section) showing focal increased FDG uptake (yellow arrow) in the distal body and fundus of the gallbladder (SUV Max: 4.3). An eccentric thickening (around 7 mm) is seen in the fundus. FDG: Fluorodeoxyglucose F 18; PET CT: Positron Emission Tomography Computed Tomography; SUV: Standardized Uptake Value.

**Figure 3:**
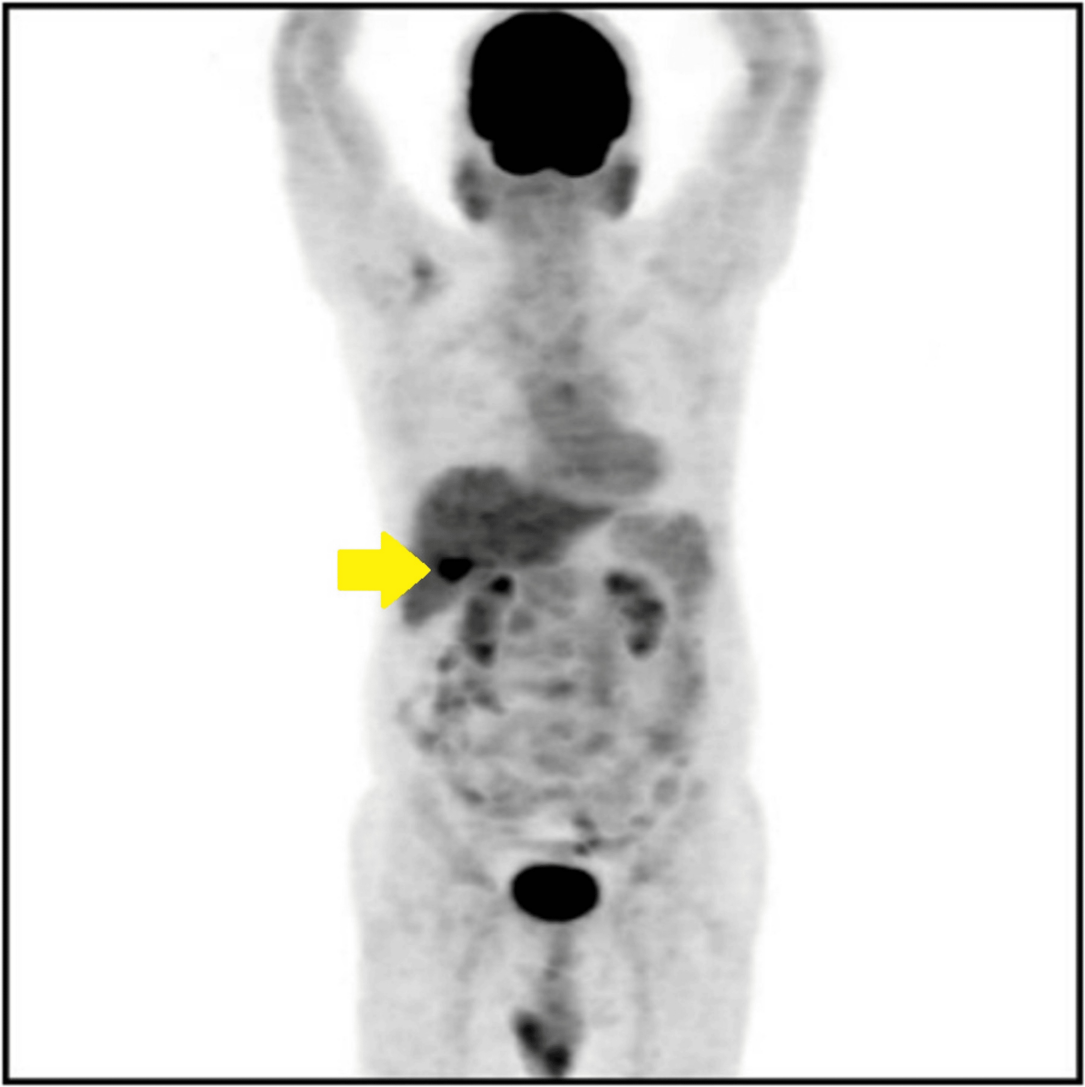
Maximum intensity projection image of a preoperative PET scan shows FDG avidity in the subhepatic region, which is the gallbladder (yellow arrow). FDG: Fluorodeoxyglucose F 18; PET: Positron Emission Tomography.

Laboratory tests revealed a normal complete blood count, electrolytes, and liver function tests. Carbohydrate antigen 19-9 (CA 19-9) was elevated at 409.8 U/mL. Ultrasound and CT scan of the abdomen (Figure 1) demonstrated a thickened gallbladder wall with a soft tissue density lesion and mild common bile duct dilatation. Fluorodeoxyglucose (FDG) positron emission tomography computed tomography (PET CT) (Figures 2-3) showed focal FDG avidity in the gallbladder fossa, suspicious for malignancy. Endoscopic ultrasound revealed a distended gallbladder with a large polypoidal mass.

Surgery and histopathology

**Figure 4:**
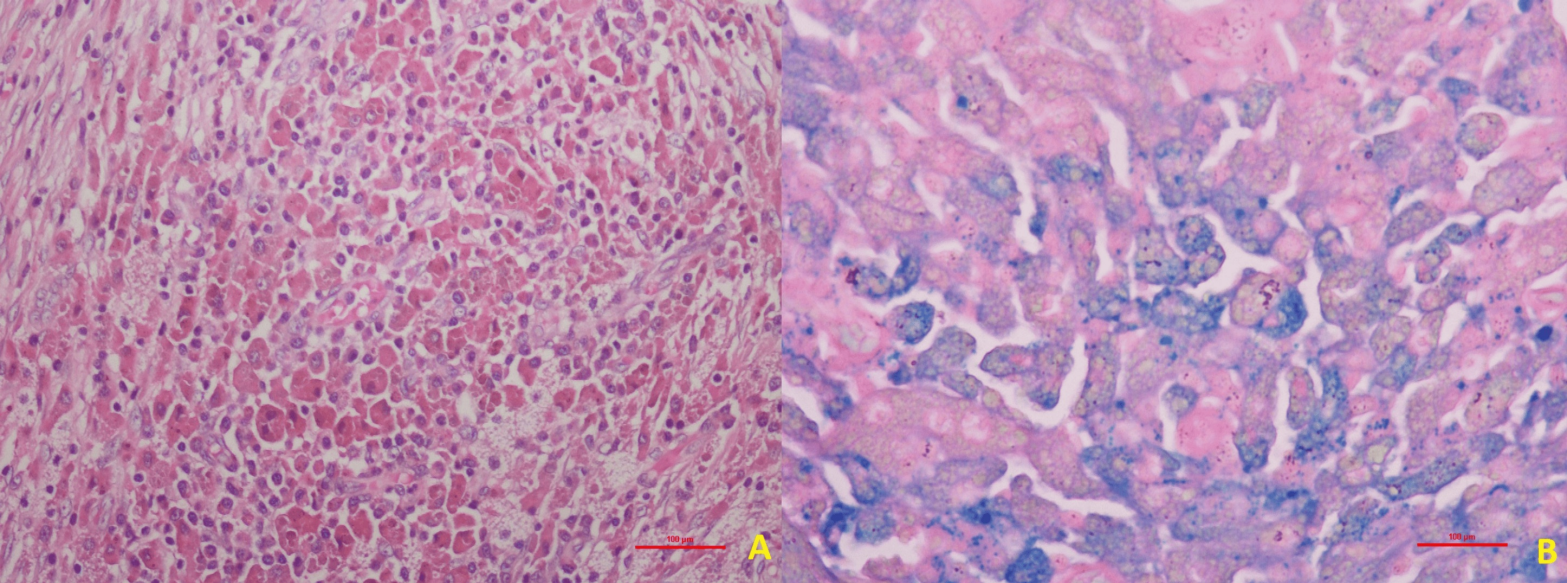
Histopathological examination (microscopy) of the gallbladder surgical specimen showing confluent sheets of histiocytes with eosinophilic granular cytoplasm and eccentrically placed nuclei (A, 100x, H&E). Iron in some of the cells is highlighted as Prussian Blue color in Perl's Stain (B, 200x, Perl's Stain).

The patient underwent an extended cholecystectomy with wedge resection of the liver. There were dense adhesions between the gallbladder and liver. On gross examination of the cut surgical specimen, a thickened gallbladder wall was noted. Histopathological microscopic examination (Figure 4) revealed a lamina propria showing moderate inflammation comprised of lymphocytes and plasma cells, and also showing fibrosis. The serosa showed dilated, congested, prominent blood vessels. The interface between the serosa of the gallbladder and liver showed sheets of histiocytes with coarse and fine granular cytoplasm. However, classical Michaelis-Gutmann bodies were not identified. On Perl's stain, many hemosiderin-laden macrophages were noted. Liver tissue and lymph nodes were unremarkable.

Postoperative course

The patient had a prolonged postoperative course due to persistent serous drainage from a subhepatic drain, likely secondary to extensive lymph node dissection. The drain was removed on postoperative day 16. He recovered well and was discharged with no fresh complaints. The patient has been under follow-up since last year and he has had no complaints.

## Discussion

Malakoplakia, a chronic inflammatory disorder, presents a diagnostic challenge due to its ability to involve various organs and mimic more common pathologies. Due to its presentation on imaging as a thickened gallbladder, the differential diagnoses include carcinoma of the gallbladder, xanthogranulomatous cholecystitis, and chronic cholecystitis [2-4].

The name, derived from the Greek words 'malakos' (soft) and 'plakos' (plaque), reflects the soft, yellow-brown plaques or nodules typically observed. On microscopy, it is characterized by sheets of histiocytes with granular cytoplasm (von Hansemann cells) and basophilic round to oval intracellular inclusions, known as Michaelis-Gutmann bodies. This is all set against a backdrop of mixed inflammatory cell infiltration. Classical Michaelis-Gutmann bodies are pathognomonic, although not necessary for diagnosis. The urinary tract is the most commonly affected site, followed by the gastrointestinal tract [1,2].

The exact cause of malakoplakia remains unclear, although it is believed to be linked to a dysfunctional immune response (as seen in HIV, immunosuppression, and cancer) to bacterial infections. The bacteria commonly implicated are E. coli, M. tuberculosis, Proteus, and S. aureus. It is postulated that in immunosuppressed states, the local macrophages are unable to digest the phagocytosed bacteria. These partially digested bacteria lead to the deposition of calcium and iron on the residual bacterial glycolipid, forming basophilic inclusion bodies called Michaelis-Gutmann bodies [1,2].

The clinical presentation of malakoplakia is highly dependent on the affected organ. In the genitourinary tract, it can manifest as recurrent urinary tract infections, dysuria, and hematuria [1]. However, as highlighted in previous case reports, the gallbladder involvement presents a unique challenge [2,5,6]. The clinical picture often mimics cholecystitis, with symptoms like recurrent abdominal pain, nausea, and vomiting. This similarity can lead to significant delays in diagnosis, as exemplified in the reviewed cases of malakoplakia. In the case report by Vaiphei K et al., the index case presented with a history of dull aching pain in the right hypochondrium associated with loss of appetite and weight [2]. Rathod SS et al. depicted two cases, where one patient experienced intermittent pain in the right upper quadrant, and the second patient presented with recurrent attacks of acute severe pain in the right hypochondrium [5]. Sajjanar AB et al. described a case with a patient having pain in the right upper quadrant of the abdomen [6].

The rarity of malakoplakia, particularly in the gallbladder, makes preoperative diagnosis difficult. Imaging studies like ultrasound, CT scans, and PET scans may be suggestive of a mass lesion, but they lack specificity [4]. The definitive diagnosis hinges on histopathological examination of the resected tissue [1,2]. In the present case report, the iron deposits inside the macrophages, noted with the help of Perl's stain on microscopy of the thickened gallbladder, are analogous to the classical Michaelis-Gutmann bodies. Otherwise, there were histiocytes with coarse and fine granular cytoplasm with surrounding inflammation comprised of lymphocytes and plasma cells, all noted on microscopy with H&E stain.

The optimal treatment strategy for malakoplakia remains debatable. Traditionally, long-term antibiotic therapy targeting the identified organism has been the mainstay of treatment [1]. However, the management of gallbladder malakoplakia presents a unique situation. Surgical resection, as performed in the reported cases, appears to be a viable option, especially in localized disease [4].

Our case report provides valuable insights into the clinical presentation, diagnostic challenges, and potential aggressiveness of gallbladder malakoplakia. The patient’s presentation with non-specific symptoms, such as recurrent epigastric pain, and the elevation of CA-19-9, a tumor marker, highlights the difficulty in differentiating this rare condition from malignancy. Histopathologic examination can help in solving this diagnostic challenge.

Moreover, the case emphasizes the potential for extensive local disease involvement in gallbladder malakoplakia, as evidenced by the patient's prolonged postoperative course due to dense adhesions and lymph node involvement. This finding suggests that aggressive surgical management may be necessary in certain cases. While the absence of identified pathogens in this instance contributes to the ongoing debate about the etiology of malakoplakia, it also highlights the need for further research into the pathogenesis of this enigmatic disease. The limitations we faced include the lack of classical Michaelis-Gutmann bodies in our case and the limited literature on this disease.

Overall, this case report expands our understanding of gallbladder malakoplakia and emphasizes the importance of considering this rare diagnosis in patients presenting with gallbladder pathology, particularly when faced with atypical clinical and radiological findings.

## Conclusions

Malakoplakia of the gallbladder is a rare but important consideration in the differential diagnosis of a thickened gallbladder wall, particularly when malignancy cannot be definitively excluded by imaging studies. Histopathological examination is essential for diagnosis. While the optimal treatment approach for gallbladder malakoplakia requires further elucidation, surgical resection appears to be a viable option, especially in cases of localized disease.
